# Assessment of Oral Masticatory Muscle Activity With Different Chewing Gums: A Cross-Sectional Study Based on Electromyogram Analysis

**DOI:** 10.7759/cureus.56849

**Published:** 2024-03-24

**Authors:** Keshav Rajesh, Sandhya Sundar, Vaishnavi Rajaraman, Ramya Ramadoss, Suresh Venugopalan

**Affiliations:** 1 Oral Pathology and Microbiology, Saveetha Dental College and Hospitals, Saveetha Institute of Medical and Technical Sciences, Saveetha University, Chennai, IND; 2 Prosthodontics and Implantology, Saveetha Dental College and Hospitals, Saveetha Institute of Medical and Technical Sciences, Saveetha University, Chennai, IND; 3 Oral Pathology and Microbiology, Saveetha Dental College and Hospitals, Saveetha Institute of Medical and Technical sciences, Saveetha University, Chennai, IND

**Keywords:** muscle activity, facial muscles, muscle of mastication, chewing gums, electromyogram

## Abstract

Background: Facial muscles, particularly those involved in mastication, play a pivotal role in the chewing process. Despite their influence on chewing, these muscles undergo alterations during mastication. Examining the relationship between chewed substances and muscle activity can provide insights into various pathological processes and aid in the development of therapeutic chewing techniques.

Aim: This study aimed to evaluate the impact of different commercially available chewing gums on the activity of key masticatory muscles.

Method: Twenty-two participants were recruited for the study. They were instructed to chew four commercially available gums: group 1 comprised sugar gum with a strong flavor; group 2 included gum containing sorbitol; group 3 consisted of gum containing xylitol; and group 4 provided sugar gum with a mild flavor. Electromyogram (EMG) recordings were utilized to assess muscle activity. Various aspects of muscle activity, including chewing time, maximum muscle potential, and coordination between different muscles, were evaluated. Data tabulation and analysis were performed using IBM SPSS software version 23.0 (IBM Corp., Armonk, NY).

Result: Analysis revealed that in terms of temporalis symmetry, group 2 exhibited the highest mean deviation, while for masseter symmetry, group 3 demonstrated the highest mean deviation. The total deviation for the temporalis and masseter muscles was 72.16% and 65.55%, respectively, indicating greater symmetry in the temporalis muscle. Additionally, group 3 displayed the highest mean deviation in both left and right-sided synergic activity of the muscles. The total deviation for the right and left sides was 64.34% and 65.67%, respectively.

Conclusion: The findings suggest that sugar-free chewing gums elicit increased muscle activity compared to sugar-containing chewing gums. Furthermore, the utilization of calorie-free chewing gums with a firm texture was associated with better-coordinated muscle activity. These results provide valuable insights into the effects of different chewing gums on masticatory muscle function and coordination, which may have implications for therapeutic interventions and oral health management.

## Introduction

Chewing gum is a popular activity enjoyed by people of all ages around the world. Besides its pleasurable sensory experience, it has been suggested to offer several potential benefits for oral health, such as reducing plaque formation, improving salivary flow, and strengthening the muscles involved in mastication [1]. However, the effects of chewing gum on oral muscle activity have not been fully elucidated, and the existing literature presents conflicting findings [2]. Electromyography (EMG) is a non-invasive method to measure the electrical activity of muscles and has been widely used to investigate the masticatory function of the jaw muscles [3,4].

Chewing is a complex physiological process that involves the coordinated action of multiple muscles in the oral cavity [5]. The masticatory muscles, which include the masseter, temporalis, medial pterygoid, and lateral pterygoid muscles, are responsible for generating the forces needed to break down food into smaller particles and mix it with saliva [6]. These muscles also play a crucial role in maintaining the stability and mobility of the jaw during chewing and other oral functions [7].

The masticatory muscles are highly specialized and adapted to the demands of chewing [8]. They are capable of producing powerful contractions that can generate forces of up to 600 N, which is essential for crushing and grinding hard and tough food items [9]. The masseter and temporalis muscles, in particular, are the primary muscles involved in the crushing and grinding of food, while the medial and lateral pterygoid muscles contribute to lateral and protrusive movements of the jaw [10].

The activity of the masticatory muscles during chewing has been extensively studied using electromyography (EMG) [11]. EMG studies have revealed that the masticatory muscles exhibit a complex pattern of activity that varies depending on the type and texture of the food being chewed. For instance, the masseter and temporalis muscles show higher activity during chewing of hard and tough foods, while the lateral pterygoid muscle shows greater activity during chewing of softer foods [12].

Understanding the role of the masticatory muscles in chewing is critical for the development of therapies for various oral disorders, including temporomandibular disorders (TMD) and malocclusion [13]. Moreover, insights gained from studying the masticatory muscles could also lead to the development of new strategies for promoting oral health and improving overall well-being [14]. This research seeks to provide insights into the impact of various chewing gums on oral muscle activity, contributing to a better understanding of their electromyographic effects. The research's null hypothesis posits that the various types of chewing gum do not exert an impact on the activity of masticatory muscles.

## Materials and methods

The study design was cross-sectional. The study was approved by the standard review board of Saveetha dental colleges and hospitals (approval no.: IHEC/SDC/UG-2116/22/DENTANAT/163). This study aims to evaluate oral masticatory muscle activity through electromyogram analysis in healthy individuals aged 18-35 who are regular consumers of chewing gum. Inclusion criteria encompass participants with a complete dentition (excluding third molars), no history of temporomandibular joint disorders or muscle-related oral pathologies, and the ability to chew gum for at least 10 minutes continuously. Exclusion criteria involve individuals with a history of temporomandibular joint disorders or masticatory muscle-related pathologies, oral or facial pain, significant dental restorations or prostheses, systemic conditions impacting muscle function, pregnancy or lactation, current use of medications affecting muscle activity, known allergies to gum ingredients, recent oral surgeries or interventions, and cognitive impairments hindering compliance with study instructions.

Each participant was provided with four different types of chewing gum: group 1 consisted of sugar gum with a strong flavor; group 2 included gum containing sorbitol; group 3 consisted of gum containing xylitol; and group 4 provided sugar gum with a mild flavor. The Food Safety and Standards Authority of India (FSSAI) approved all chewing gums used in the study, and local shops provided them. Prior to experimentation, all gum batches were stabilized. The chewing duration for each gum was standardized to two minutes, with participants alternating sides while wearing EMG electrodes. A one-minute rest interval was provided between each gum type. Importantly, participants were unaware of the specific type of chewing gum they were assigned to chew, ensuring a blinded experimental setup.

**Figure 1:**
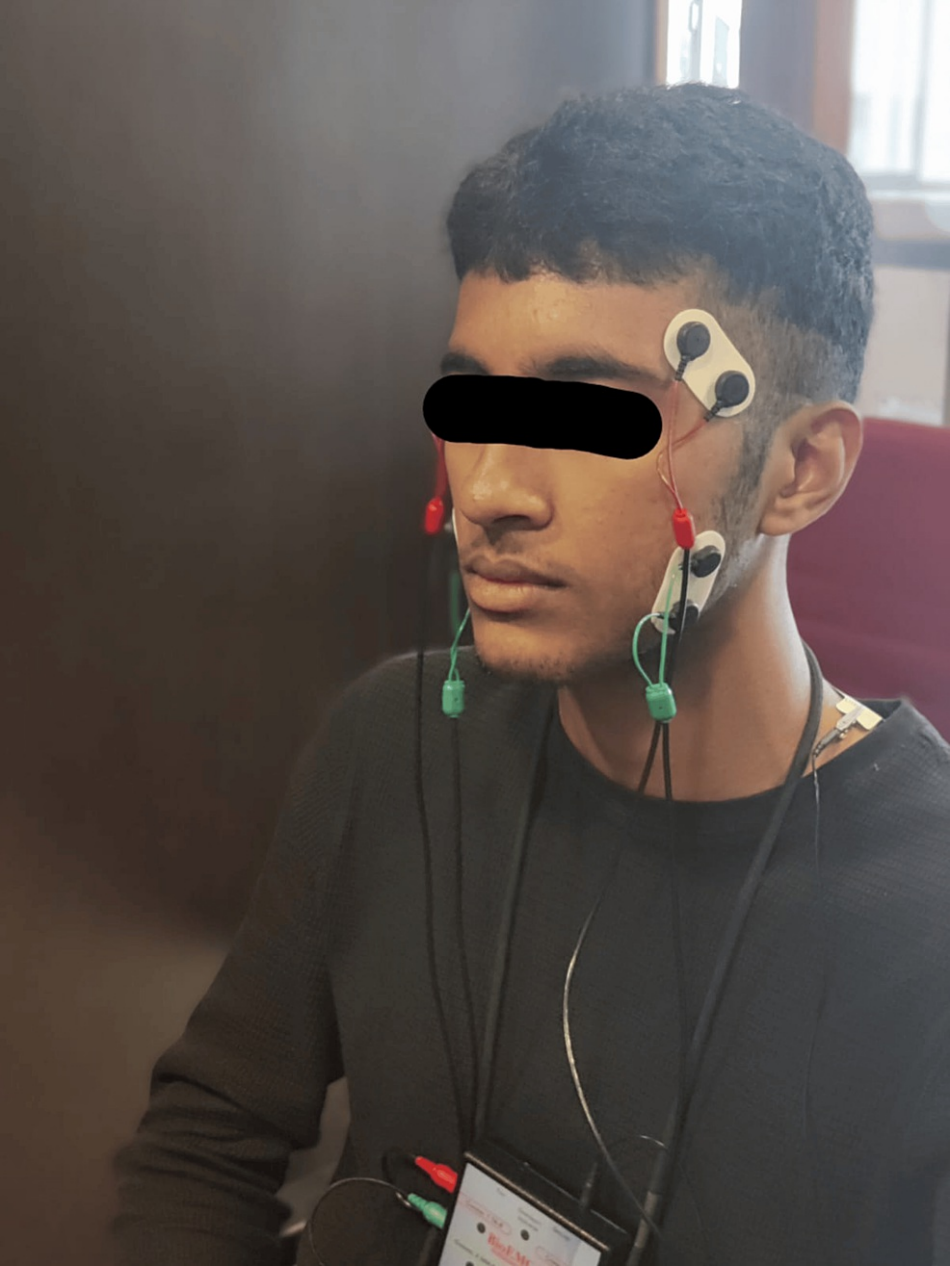
The electrodes were placed on the subject as seen in the picture

For recording the muscle activity, the EMG electrodes (Bio Research®, Milwaukee, WI, USA) were carefully positioned over the masseter muscle (anterosuperior to the angle of the mandible) and the temporalis muscle (above a line drawn from the upper earline to the canthus of the eye), maintaining an interelectrode distance of 8 mm, as shown in Figure 1. The EMG signals were recorded using a 16-channel portable EMG system. The raw EMG signals were bandpass filtered, rectified, and smoothed using a root mean square algorithm and Bio-Pak software. The EMG activity was expressed as a percentage of the maximum voluntary contraction (MVC) obtained during a maximum clenching task. The baseline activity of the participant's masticatory muscles was recorded as control using the same EMG setup.

The EMG data for each type of chewing gum, focusing on both the masseter and temporalis muscles, underwent analysis. These data were organized into tables and subjected to statistical analysis using IBM SPSS software version 23 (IBM Corp., Armonk, NY). The statistical method employed was a one-way analysis of variance (ANOVA), with a significance level set at a 95% confidence interval.

## Results

**Figure 2:**
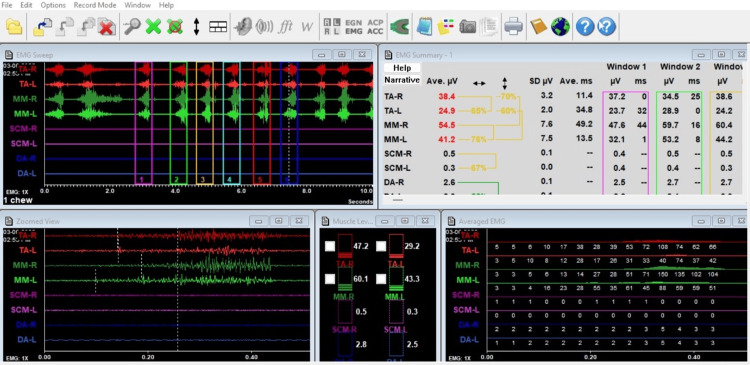
Electromyographic reading of group 2 showing 65% and 76% of symmetry between right and left temporalis and masseter, respectively. The synergy in right side and left side muscles were 70% and 60%, respectively.

A total of 22 healthy adults (11 males and 11 females) were recruited in the study, with their average ages in the range of 19±2.5 years. An electromyogram (EMG) was utilized to evaluate the muscle activity, synergy, and symmetry of the masseter and temporalis muscles, an example of which is shown in Figure 2.

The symmetry between the right side (RT) and left side (LT) temporalis and masseter muscles using different chewing gums (groups 1-4) was measured with EMG. The mean symmetry produced by group 1 chewing gums in temporalis was 75.32% ± 13.877, and for masseter muscle, it was 66.89% ± 14.712. The mean symmetry produced by group 2 chewing gums in temporalis was 79.05% ± 12.651, and for masseter muscle, it was 67.11% ± 19.189. For groups 3 and 4, the mean symmetry for the temporalis muscle was 74.00% ± 17.375 and 70.26% ± 17.931, respectively, and for the masseter muscle, it was 73.58% ± 19.651 and 66.37% ± 24.902, respectively. The mean symmetry produced by control chewing gums in temporalis and masseter muscles was 62.16% ± 31.907 and 53.79% ± 31.648, respectively. The total deviation for temporalis was 72.16%, and the total deviation for masseter was 65.55%. When a one-way ANOVA was performed, no statistical difference between the study groups was observed (p > 0.05) (Table 1).

**Table 1:** The mean symmetry between temporalis and masseter muscles for the study groups assessed using one way ANOVA. TA: temporalis, MM: masseter.

		Mean	Standard deviation	P-value
TA symmetry	Group 1	75.32%	13.877	0.108
	Group 2	79.05%	12.651	
	Group 3	74.00%	17.375	
	Group 4	70.26%	17.931	
	Control	62.16%	31.907	
	Total	72.16%	20.374	
MM symmetry	Group 1	66.89%	14.712	0.117
	Group 2	67.11%	19.189	
	Group 3	73.58%	19.651	
	Group 4	66.37%	24.902	
	Control	53.79%	31.648	
	Total	65.55%	23.204	

While comparing the synergy on the RT, it was noted that the synergy on the RT muscles in groups 1-4 and control were 67.26% ± 15.506, 66.21% ± 17.290, 67.58% ± 16.101, 66.16% ± 16.503, and 54.47% ± 31.652, respectively. There was no statistically significant difference between the study groups. In regards to the LT muscles, the mean synergy for groups 1-4 and control was 70.21% ± 16.019, 68.32% ± 21.232, 67.47% ± 17.859, 65.68% ± 22.723, and 56.68% ± 30.481, respectively, with no statistically significant difference. The total deviation of the RT was 64.34%, and the total deviation of the LT was 65.67% (Table 2).

**Table 2:** The mean synergy between right side and left side, on using the different groups of chewing gum as assessed using one way ANOVA. RT: right side, LT: left side

		Mean	Standard deviation	P-value
RT synergy	Group 1	67.26%	15.506	0.237
	Group 2	66.21%	17.290	
	Group 3	67.58%	16.101	
	Group 4	66.16%	16.503	
	Control	54.47%	31.652	
	Total	64.34%	20.538	
LT synergy	Group 1	70.21%	16.019	0.375
	Group 2	68.32%	21.232	
	Group 3	67.47%	17.859	
	Group 4	65.68%	22.723	
	Control	56.68%	30.481	
	Total	65.67%	22.268	

## Discussion

Chewing is a vital part of the digestive process, involving the masticatory muscles, including the masseter and temporalis muscles. Chewing gum stimulates masticatory muscles through repeated compression and relaxation cycles, generating a rhythmic stimulus. This stimulates the masseter and temporalis muscles, leading to increased muscle contraction and activity. The continuous and repetitive movement pattern encourages sustained muscle engagement, promoting endurance and strength development. The distribution of masticatory strikes across both sides of the jaw promotes uniform muscle activation, mitigating asymmetries [15]. Proprioceptive feedback modulates muscle activity, allowing precise coordination and control. Chewing gum can positively impact masticatory muscle performance and health [16].

The present study used EMG to investigate the effect of four different chewing gums on masticatory muscle activity. This variety allows for investigating potential differences in muscle activity and response to different sensory stimuli. By including a variety of gum types, the study can examine how different ingredients, such as flavorings, sugars, and additives, impact muscle activity during chewing. The selection of these gum types may also reflect their prevalence in the market and relevance to everyday consumer habits.

The study findings demonstrated notable variations in masticatory muscle activity depending on the type of chewing gum. Despite observing variations in mean symmetry values across different groups and muscles, the absence of statistical significance implies that these differences may not have been substantial enough to definitively establish a significant distinction between the groups. While there was consistency across groups, suggesting that the type of chewing gum might not have exerted a significant overall effect on muscle activity symmetry, it is important to note that variations were still present. The high standard deviations within each group suggest considerable variability, which could have contributed to the lack of statistically significant differences between groups. Additionally, the small sample size may have limited the study's ability to detect potentially meaningful differences between groups, despite the potential influence of different types of chewing gum on muscle activity.

Notably, the sugar-free gums (groups 2 and 3) elicited higher muscle activity compared to the sugar-containing gum types (groups 1 and 4) and the control. The sugar-free chewing gums not only reduced plaque quantity and gingival inflammation but also had a positive impact on the chewing cycle [17]. The evidence suggests that sugar-free chewing gum has a caries-reducing effect, possibly due to saliva stimulation, a lack of sucrose, and the inability of bacteria to metabolize polyols into acids [18]. These factors extend the chewing duration, resulting in sustained muscle activation and increased muscle activity, as captured by the EMG.

Sugar-free gums typically have a firmer texture and greater resistance compared to sugar-containing gums. The increased resistance during chewing necessitates stronger muscle contractions, leading to higher muscle activity detected by EMG [19]. This increased resistance could lead to higher muscle activity compared to softer-textured gums. A study by Matsuo et al. found that hard-textured foods increased muscle activity in all age groups, particularly in older adults with oral hypofunction. This suggests that these foods could enhance masticatory load and muscle activity, thereby improving overall health [20]. Another explanation could be that the hardness of the gum stimulates the proprioceptors in the oral cavity, which in turn activate the masticatory muscles [21].

The finding that strong-flavored sugar gum induces greater muscle activity compared to milder alternatives is intriguing. While chewing itself is the primary stimulant for the masticatory muscles, flavor can also influence their activity to some degree. Intense flavors may trigger heightened salivary gland activity, increasing saliva production and promoting more effective mastication [22]. Additionally, strong flavors may serve as sensory stimuli, eliciting a robust motor response from the masticatory muscles [23].

The present study acknowledges several limitations. First, due to a limited sample size, it did not explore the influence of gender on the electromyographic effects of chewing gum. Second, it did not examine the long-term effects of gum chewing on masticatory muscle function or oral health. Future studies could mitigate these limitations by incorporating a more diverse sample and investigating the effects of long-term gum chewing on masticatory muscle activity and oral health outcomes.

The results of this study have practical implications for the development of chewing gum products that can promote better masticatory muscle activity and oral health. The finding that mint-flavored gum produced higher muscle activity suggests that adding mint flavor to gum products could enhance their efficacy. The finding that hard gum produced the highest muscle activity suggests that chewing harder gum may promote better masticatory muscle activity.

## Conclusions

This study used EMG to examine how different chewing gums affected the activation of the masticatory muscles. Therapeutic gums, followed by mint-flavored gums, had the highest stimulatory activity on masticatory muscles. The research contributes to the body of knowledge on chewing gum products and masticatory muscle activity. The results emphasize how crucial it is to choose the correct kind of chewing gum in order to improve oral health and masticatory muscle activation. The long-term impacts of chewing gum on masticatory muscle performance and dental health outcomes require more investigation.
